# Brazilian Red Propolis Is as Effective as Amoxicillin in Controlling Red-Complex of Multispecies Subgingival Mature Biofilm In Vitro

**DOI:** 10.3390/antibiotics9080432

**Published:** 2020-07-22

**Authors:** Kadmo Azevedo de Figueiredo, Helio Doyle Pereira da Silva, Stela Lima Farias Miranda, Francisco Jerfeson dos Santos Gonçalves, Arlene Pereira de Sousa, Luciene Cristina de Figueiredo, Magda Feres, Bruno Bueno-Silva

**Affiliations:** Dental Research Division, Guarulhos University, Guarulhos, São Paulo, Guarulhos 07023-070, Brazil; kadmodonto@hotmail.com (K.A.d.F.); heliodoyle@yahoo.com.br (H.D.P.d.S.); stelalima.odonto@hotmail.com (S.L.F.M.); francisco.jerfeson@outlook.com (F.J.d.S.G.); arlenesousa1@gmail.com (A.P.d.S.); lucienedefigueiredo@gmail.com (L.C.d.F.); mferes@ung.br (M.F.)

**Keywords:** propolis, antimicrobial, periodontal disease, amoxicillin

## Abstract

This study investigated the effects of Brazilian Red Propolis (BRP) extract on seven-day-old multispecies subgingival biofilms. Mixed biofilm cultures containing 31 species associated with periodontal health or disease were grown for six days on a Calgary device. Then, mature biofilms were treated for 24 h with BRP extract at different concentrations (200–1600 µg/mL), amoxicillin (AMOXI) at 54 µg/mL (positive control) or vehicle (negative control). Biofilm metabolic activity was determined by colorimetry, and bacterial counts/proportions were determined by DNA–DNA hybridization. Data were analyzed by Kruskal–Wallis and Dunn’s tests. Treatment with BRP at 1600, 800 and 400 μg/mL reduced biofilm metabolic activity by 56%, 56% and 57%, respectively, as compared to 65% reduction obtained with AMOXI. Mean total cell counts were significantly reduced in all test groups (~50–55%). Lower proportions of red, green and yellow complex species were observed upon treatment with BRP (400 µg/mL) and AMOXI, but only AMOXI reduced the proportions of *Actinomyces* species. In conclusion, BRP extract was as effective as AMOXI in killing seven-day-old multispecies biofilm pathogens and did not affect the levels of the host-compatible *Actinomyces* species. These data suggest that BRP may be an alternative to AMOXI as an adjunct in periodontal therapy. In vivo studies are needed to validate these results.

## 1. Introduction

The main etiological factor of periodontal disease is a dysbiosis of the oral subgingival biofilm associated with the presence of periodontopathogens, mainly *Porphyromonas gingivalis*, *Tannerella forsythia*, *Treponema denticola* and *Aggregatibacter actinomycetemcomitans*. These microorganisms release metabolic byproducts or components (e.g., lipopolysaccharides and fimbriae) into periodontal tissues, causing an exacerbated inflammatory response which leads to tissue destruction [1]. Periodontal diseases are estimated to affect between 20 and 50% of the world population, with a negative impact on the individual’s health-related quality of life [2].

The treatment of periodontal diseases consists of controlling the occurrence of periodontopathogenic microorganisms to reduce or eliminate tissue inflammation. Mechanical removal of subgingival biofilms by means of scaling and root planning (SRP) significantly improves all periodontal clinical parameters. [3]. However, the mechanical therapy alone may not be fully effective to reverse the major dysbiosis associated with periodontitis, requiring the use of systemic antibiotics. Systemic administration of amoxicillin (AMOXI) was shown to be an effective adjunctive treatment in the management of periodontitis [4,5]. In addition, the combination of AMOXI and metronidazole (MTZ) with SRP has proven an effective approach [6,7,8]. However, some essential questions associated with the use of these antibiotics remain unanswered, such as potential systemic side effects and the risk of selecting resistant strains [9].

Natural products, such as propolis, have attracted the interest of researchers and laypeople worldwide due to the recent scientific evidence of their beneficial pharmacological properties [10,11]. Brazilian Red Propolis (BRP) has a unique chemical composition that differs from that of other types of Brazilian propolis. BRP contains mostly polar compounds, which facilitates its dissolution in aqueous vehicles and sustains the presence of several isoflavones—compounds with documented beneficial properties for humans [12]. The main relevant pharmacological actions of BRP include antimicrobial [13,14,15], anti-inflammatory [16,17,18], anti-tumoral [19], healing [20], antioxidant and antiparasitic properties [15].

Recently, our research group reported that BRP extract is effective in reducing multispecies subgingival biofilm formation [13], but its effects on mature biofilms remain to be determined. Disruption of mature biofilms by antimicrobial agents is challenging and commonly ineffective since mature biofilm cultures are more virulent than newly formed ones or than planktonic cells [21,22]. Thus, in this study, we investigated the inhibitory effects of BRP extract on mature multispecies subgingival biofilms comparatively to AMOXI. 

## 2. Results

**Figure 1:**
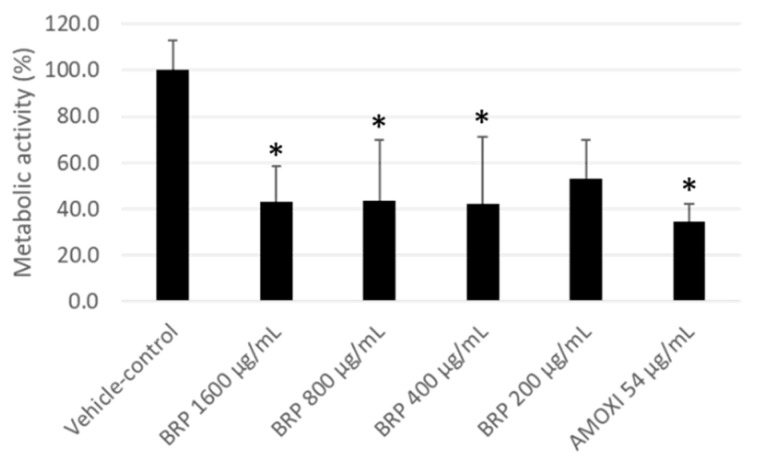
Metabolic activity of multispecies biofilm cultures treated with the dilution vehicle (negative control), Brazilian Red Propolis (BRP) ethanolic extract (1600, 800, 400 and 200 µg/mL) and amoxicillin (AMOXI) (54 µg/mL). Vehicle-treated biofilms were considered as with 100% metabolic activity. (*) indicates significant differences when compared to the control group (Kruskal–Wallis followed by Dunn’s post-hoc test, *p* ≤ 0.05).

Figure 1 shows the effects of BRP extract and controls on the metabolic activity of multispecies biofilm cells. Treatment with BRP extract at 1600, 800 and 400 μg/mL significantly reduced biofilm metabolic activity by 56%, 56% and 57%, respectively, as compared to the vehicle control (*p* < 0.05). However, there was no significant difference in metabolic activity between cultures treated with BRP at 200 μg/mL and the vehicle (*p* > 0.05).

**Figure 2:**
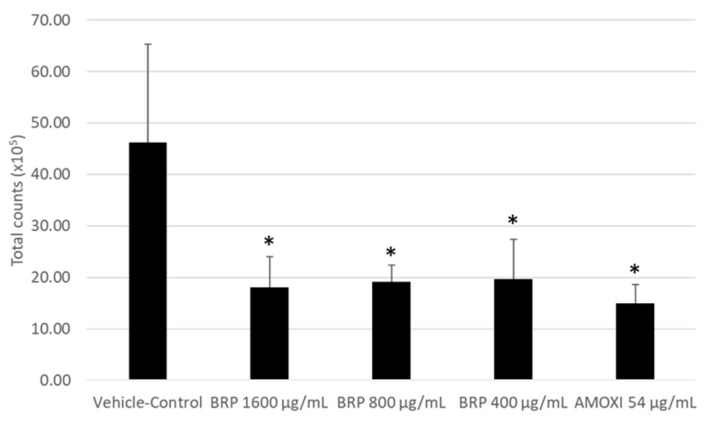
Total bacterial counts (× 10^5^) in biofilms treated with the BRP ethanolic extract at 1600, 800 and 400 µg/mL and amoxicillin (AMOXI) at 54 µg/mL. (*) indicates a significant difference when compared to the control group (Kruskal–Wallis followed by Dunn’s post-hoc test, *p* ≤ 0.05).

Since the extract concentration of 200 μg/mL did not reduce biofilm metabolic activity, we did not include this treatment group in the checkerboard DNA–DNA hybridization analysis. Figure 2 shows the mean total counts of biofilm cells treated with BRP (1600, 800 and 400 μg/mL) and AMOXI (54 μg/mL). All treatment groups were different from the vehicle control (*p* < 0.05), with no significant difference between them (*p* > 0.05). Interestingly, no significant difference in total cell counts was observed between biofilm cultures treated with BRP extract, regardless of the tested concentration, and those treated with the standard drug AMOXI (*p* > 0.05).

**Figure 3:**
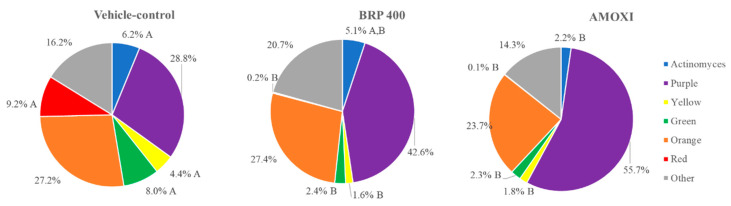
The effects of Brazilian Red Propolis (BRP) extract, amoxicillin (AMOXI) and vehicle control on the proportion of bacterial complexes. The colors represent different microbial complexes as described by Socransky et al., 1998. The data were analyzed by Kruskal–Wallis followed by Dunn’s post-hoc test. Different letters indicate significant differences between groups within the same bacterial complex (*p* ≤ 0.05).

As there was no statistical difference between the three tested concentrations of BRP extract, the lowest effective concentration was selected for further analysis. Figure 3 shows that treatment with BRP (400 µg/mL) and AMOXI (54 μg/mL) significantly reduced the proportions of red-complex (disease-associated), green- and yellow-complex (health-associated) bacterial strains and that only AMOXI decreased the proportions of *Actinomyces* species, as compared to the control group (*p* < 0.05).

**Figure 4:**
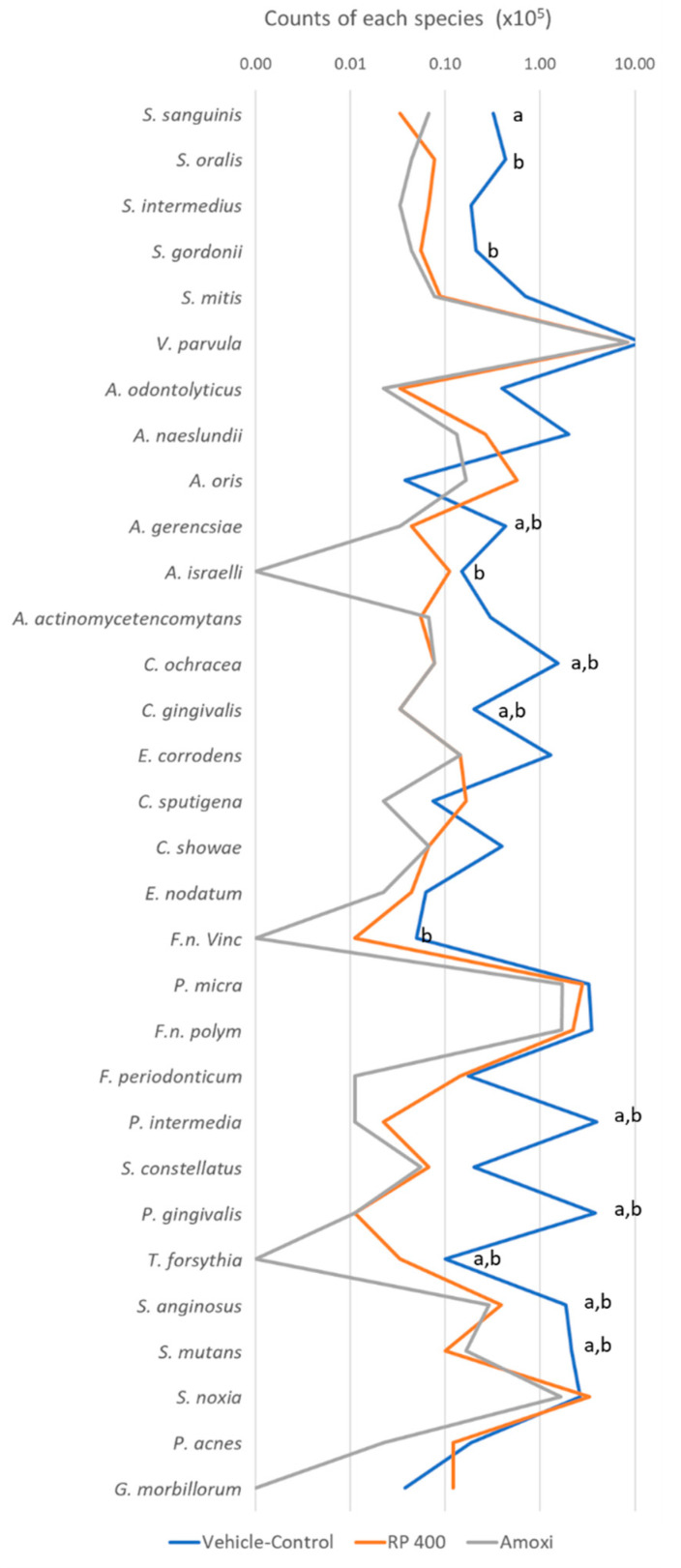
Mean total counts of bacterial strains in biofilms treated with the vehicle control, BRP extract (400 μg/mL) and amoxicillin (AMOXI) (54 μg/mL). The data were analyzed by Kruskal–Wallis followed by Dunn’s post-hoc test (*p* < 0.05). Letter “a” indicates statistically significant differences between BRP (400 μg/mL) and the vehicle-control but no difference between BRP (400 μg/mL) and AMOXI; letter “b” indicates statistically significant differences between AMOXI and the vehicle-control but no difference between AMOXI and BRP (400 μg/mL).

Figure 4 shows the mean total counts of each bacterial strain in treated biofilms. Treatment with BRP (400 µg/mL) and AMOXI (54 μg/mL) reduced the mean counts of eight different species (*Actinomyces gerencseriae*, *Capnocytophaga ochracea*, *Capnocytophaga gingivalis*, *Prevotella intermedia*, *Porphyromonas gingivalis*, *Tannerella forsythia*, *Streptococcus anginosus* and *Streptococcus mutans*) when compared to vehicle-treated biofilms (*p* < 0.05). *Streptococcus sanguinis* counts were significantly reduced only in BRP-treated biofilms, whereas *Streptococcus oralis*, *Streptococcus gordonii*, *Actinomyces israelii* and *Fusobacterium nucleatum vincentii* counts were reduced only in AMOXI-treated biofilms, as compared to the control group (*p* < 0.05).

## 3. Discussion

Our study showed that BRP extract was effective in reducing the metabolic activity and total cell counts of red-complex strains in mature biofilms similarly to AMOXI. Moreover, both substances significantly reduced the mean counts of eight bacterial species, including well-known periodontal pathogens, such as *P. gingivalis*, *T. forsythia* and *P. intermedia*, while the proportions of the host compatible *Actinomyces* species were only reduced by AMOXI.

Subgingival biofilms associated with periodontitis are classically categorized into color-coded complexes, according to their role in periodontal health or disease. The red complex is composed by microorganisms associated with disease; the orange complex is associated with the health–disease transition, while the yellow, purple and green complexes as well as *Actinomyces* species are associated with a healthy periodontal condition [23]. It has been well established that pathogens in the red and orange complexes may trigger and/or sustain the dysbiosis state in biofilms associated with periodontitis, thereby leading to a persistent, exacerbated and damaging inflammatory response [1]. Thus, the effects of BRP in reducing the proportion of red-complex pathogens, such as *P. gingivalis* and *T. forsythia*, similarly to a potent antibiotic such as AMOXI, was considered a remarkable result.

A recent study showed that BRP extract was able to reduce the proportions of red and orange bacterial complexes at the concentration of 1600 μg/mL, while chlorhexidine reduced only those from the red complex [13]. The differences between our findings and those of Miranda et al. [13] can be explained by the treatment regimen used in our studies. While Miranda et al. [13] performed two 1 min daily treatments during biofilm formation, we grew biofilm cultures for six days to a mature state and then treated them for 24 h. This protocol was selected to mimic the systemic use of antibiotics, which are normally administered for one/two week(s). Although mature biofilms are more resistant to antimicrobials than immature biofilms, the effective concentration of BRP extract for a 24 h treatment (400 μg/mL) was four-fold lower than that used for daily treatments (1600 μg/mL) [13]. Once the contact period of the BRP extract with biofilm cells was longer in our study, we reasoned that testing a lower effective concentration of the extract would be more appropriate, particularly because our findings showed that increasing BRP extract concentration above 400 μg/mL does not augment the inhibitory effects of the extract against mature biofilms.

An intriguing result observed in our study was that AMOXI-treated biofilms showed lower proportions of beneficial *Actinomyces* species. Interestingly, a previous clinical study showed a considerable decrease in the proportions of *Actinomyces* species up to one year after AMOXI administration in a group of adults with periodontitis. This may be considered an undesired effect of AMOXI since species of the genus *Actinomyces* have been closely related to rebiosis [4,6,24].

The combination of AMOXI and MTZ with SRP has been well studied and has shown clinically relevant benefits for the treatment of severe periodontitis and killing of subgingival pathogens [6]. However, another study using the same in vitro subgingival biofilm model showed that administration of AMOXI alone or AMOXI plus MTZ for a period of 24 h has similar effects on biofilm metabolic activity [25]. Therefore, in the present study, we used only AMOXI as a positive control. The concentration of AMOXI (54 μg/mL) used in our study was the most effective one from a range of concentrations previously tested in a 24 h treatment [25]. Consistent with the findings reported by Soares et al., our study showed a very similar effectiveness of AMOXI in reducing biofilm metabolic activity by 65%.

While systemic antibiotics are clinically effective, there have been reports of side effects [7] and, more importantly, a significant increase in antibiotic resistance rates worldwide [26]. Altogether, this scenario has encouraged researchers to constantly seek novel candidates for co-adjuvant periodontal therapy, such as propolis, probiotics and resolvins [27,28,29,30]. The interplay between microbial dysbiosis and inflammation on the onset and progression of periodontal diseases has been a topic of debate, and apparently, BRP extract seems to have effectivity on both aspects. It has been recently shown in vitro and in vivo that BRP can modulate the inflammatory process by inhibiting the Toll-like response and the NF-κB pathway, an important inflammatory mediator [16,17,18,31]. In addition, our findings and others reported elsewhere [13] indicate that BRP extract has an excellent killing effect against periodontal pathogens during biofilm formation. These data suggest that BRP holds concomitant anti-inflammatory and antibacterial properties. Its complex chemical composition, including the presence of formonetin, medicarpin, neovestitol and vestitol, may explain the major antimicrobial and anti-inflammatory properties displayed by the extract [14,32].

Isolating and purifying fractions from natural products is a relevant and novel strategy in drug discovery and development. Working with crude extracts may prove a challenging task due to their complex chemical composition and the common presence of compounds with unknown polarity and solubility [33]. A fraction containing both neovestitol and vestitol, which are compounds isolated from BRP, showed significant activity against *Streptococcus mutans* biofilms [34]. Thus, the neovestitol/vestitol fraction of BRP should be further tested in subgingival biofilm models. Additionally, these compounds were shown to have anti-inflammatory properties when tested isolated. Both neovestitol and vestitol were reported to modulate the NF-κB pathway in lipopolysaccharide (LPS)-activated macrophages [35,36] and decreased neutrophil migration, rolling and adhesion, by reducing the expression of ICAM-1 in an in vivo LPS-induced acute peritonitis model [37,38]. Lastly, a recent study also showed that these compounds downregulated the expression of proteins commonly used as targets for cancer treatment [39].

Despite the body of evidence showing the beneficial properties of BRP, its clinical use still warrants further research. Since most of the published articles on this topic report laboratorial or animal studies, the safety and possible toxicity of BRP in humans remain to be determined. 

## 4. Materials and Methods

### 4.1. Preparation of BRP Extract

BRP samples were collected in a private farm in the city of Maceio, Alagoas State, northeastern Brazil. The samples were scraped off the boxes in which they were transported, which also contained *Apis mellifera* bees. Any residues of bee wax or other materials were removed. The chemical analysis of BRP samples was previously performed by our research group [16]. High-performance liquid chromatography revealed the main compounds found in BRP samples were formononetin, vestitol, neovestitol, quercetin, liquiritigenin and isoliquiritigenin [16], which is in accordance with the literature [14,15,31]. Briefly, the BRP ethanolic extract was obtained by adding 25 g of red propolis into 200 mL of 80% ethanol/ 20% water (*v/v*) under continuous mixing for 45 min. Next, the suspension was filtered with qualitative filter paper (80 g) to remove any possible impurity, the solvent was evaporated using a rotary evaporator equipment, and the BRP ethanolic extract was obtained, with a yield of 73% (from 100 g of red propolis, 73 g of BRP ethanolic extract was obtained). The extract was kept at 4 °C and protected from light to prevent stability loss. The BRP ethanolic extract was re-diluted in 80% ethanol to obtain concentrations of 3200, 1600, 800 and 400 µg/mL. Prior to biofilm treatments, the BRP extracts were diluted 1:1 with the culture media, with final treatment concentrations as 1600, 800, 400 and 200 µg/mL. The final ethanol concentration in the extract used in the treatment was 3.2%.

### 4.2. In Vitro Multispecies Biofilm Model

In vitro multispecies biofilm cultures were prepared as described by Miranda et al. [13], with some modifications. The bacterial species included in the biofilm model are listed in Table 1.

Tryptone soy agar with 5% sheep blood (Probac, São Paulo, Brazil) was used to grow most species under anaerobic conditions, 85% nitrogen, 10% carbon dioxide and 5% hydrogen, while *Eubacterium nodatum* were cultured on fastidious anaerobic agar with 5% sheep blood. *Porphyromonas gingivalis* was grown on tryptone soy agar containing yeast extract enriched with 1% hemin, 5% menadione and 5% sheep blood. *Tannerella forsythia* was grown on tryptone soy agar containing yeast extract enriched with 1% hemin, 5% menadione, 5% sheep blood and 1% *N*-acetylmuramic acid. All species were allowed to grow on agar plates for 24 h and then transferred to glass tubes containing BHI culture medium (Becton Dickinson, Sparks, MD, USA) supplemented with 1% hemin. After 24 h growing on conical tubes, the optical density (OD) was adjusted for the inoculum to have about 10^8^ cells/mL of each species. A dilution of individual cell suspensions was performed, and 100 µL aliquots containing 10^6^ cells from each species were added to 11,700 µL of BHI broth complemented with 1% hemin and 5% sheep blood to obtain an inoculum of 15 mL.

The multispecies biofilm model was developed using a Calgary biofilm device (CBD) in a 96-well plate (Nunc; Thermo Scientific, Roskilde, Denmark). A 150 µL aliquot of each inoculum was added to the wells and corresponded to ~1 × 10^4^ cells of each bacterial strain, except for *P. gingivalis* and *Prevotella intermedia*, whose inocula were adjusted to 2 × 10^4^ cells. A lid containing polystyrene pins was used to seal the 96-well plate (Nunc TSP system; Thermo Scientific, Roskilde, Denmark). Coated plates were incubated at 37 °C under anaerobic conditions. On day three, the spent medium (BHI broth with 1% hemin and 5% sheep blood) was replaced, and biofilm cultures were kept at 37 °C under anaerobic conditions for an additional four days to obtain seven-day-old biofilms [13].

### 4.3. Treatments with BRP Extract

Biofilm cultures were treated with the BRP extract for 24 h after six days of biofilm development. Biofilm-coated CBD pins were transferred to 96-well plates containing culture media plus the BRP ethanolic extract (1600, 800, 400 or 200 µg/mL), dilution vehicle (negative control) and AMOXI at 54 μg/mL (positive control). All groups were diluted with culture media at a 1:1 ratio. The vehicle control was the same solution used to dissolve the BRP extract and consisted of 6.4% ethanol in 10% phosphate buffer (*v*/*v*, final concentration: 3.2%). After treatment, the pins were washed with PBS and submitted to biological tests, as following described.

### 4.4. Quantification of Biofilm Metabolic Activity

The effects of BRP extract and controls on the metabolic activity of multispecies biofilm cells were measured in a spectrophotometric assay with 2,3,5-triphenyltetrazolium chloride (TTC) (catalog No. 17779; Fluka analytical). TTC is used to differentiate between metabolically active and inactive cells. TCC white substrate is enzymatically reduced to red formazan 1,3,5-triphenyl by live cells due to the activity of several dehydrogenases. The change in substrate color is an indirect measure of bacterial metabolic activity.

To measure the metabolic activity of biofilm cells, the pins were transferred to 96-well plates with 200 μL/well of fresh BHI medium supplemented with 1% hemin and 0.1% TTC solution. The plates were incubated under anaerobic conditions for 8 h at 37 °C. TTC reduction to red formazan was read at 485 nm in a spectrophotometer [13].

### 4.5. Checkerboard DNA–DNA Hybridization

The pins coated with seven-day-old biofilms from each group were transferred to Eppendorf tubes containing 100 μL of TE buffer (10 mM Tris-HCl, 1 mM EDTA (pH 7.6)); then, 100 μL of 0.5 M NaOH was added to each tube. The tubes containing the pins and the final solution were boiled for 10 min, and the solution was neutralized by adding 0.8 mL of 5 M ammonium acetate. The samples were individually analyzed for the presence and count of the 31 bacterial species using the DNA–DNA hybridization technique, as previously described (Socransky et al., 1994; Mestnik et al., 2010). Briefly, following sample lysis, the DNA was placed onto a nylon membrane using a Minislot device (Immunetics, Cambridge, USA) and fixed onto the membrane at 120 °C for 20 min. Next, the membrane was placed in a Miniblotter 45 (Immunetics). Digoxigenin-labelled whole genomic DNA probes of the 31 bacterial species were hybridized in each lane of the Miniblotter. Following hybridization, the membranes were washed, and DNA probes were detected using a specific antibody to digoxigenin conjugated with phosphatase alkaline. The signals were detected using AttoPhos substrate (Amersham Life Sciences, Arlington Heights, USA), and the data were obtained in Typhoon Trio Plus program (Molecular Dynamics, Sunnyvale, USA). Two lanes in each membrane contained the standards with 1 × 10^5^ and 1 × 10^6^ cells of each strain. The signals were converted into absolute counts via comparison with the standards on the same membrane. Failure to detect a signal was recorded as zero. The measurements of the experimental groups were compared against those of the negative and positive controls. Counts below the method detection limit (1 × 10^4^) were considered zero [40].

### 4.6. Statistical Analysis

The metabolic activity data were analyzed by Kruskal–Wallis followed by Dunn’s post-hoc test, whereas the microbial composition (checkerboard DNA–DNA hybridization) data were analyzed using Kruskal–Wallis followed by Dunn’s post-hoc test. A 5% significance level was considered in all statistical tests.

## 5. Conclusions

Collectively, our findings showed that BRP (at a minimal concentration of 400 μg/mL) extract was as effective as 54 μg/mL of AMOXI in killing seven-day-old multispecies biofilm pathogens and did not affect beneficial *Actinomyces* spp. growth. This suggests that BRP may be an alternative to AMOXI as an adjunct in periodontal therapy, but in vivo studies are needed to validate these results.

## Figures and Tables

**Table 1:** List of bacterial species cultured in multispecies biofilms. The strains were categorized into the microbial complexes described by Socransky et al. [25].

Multispecies Biofilm Strains
Actinomyces complex
*Actinomyces naeslundii* ATCC 12104
*Actinomyces oris* ATCC 43146
*Actinomyces gerencseriae* ATCC 23840
*Actinomyces israelii* ATCC 12102
Purple complex *Veillonella parvula* ATCC 10790
*Actinomyces odontolyticus* ATCC 17929
Yellow complex *Streptococcus sanguinis* ATCC 10556
*Streptococcus oralis* ATCC 35037
*Streptococcus intermedius* ATCC 27335
*Streptococcus gordonii* ATCC 10558
*Streptococcus mitis* ATCC 49456
Green complex *Aggregatibacter actinomycetemcomitans* ATCC 29523
*Capnocytophaga ochracea* ATCC 33596
*Capnocytophaga gingivalis* ATCC 33624
*Eikenella corrodens* ATCC 23834
*Capnocytophaga sputigena* ATCC 33612
Orange complex *Campylobacter showae* ATCC 51146 *Eubacterium nodatum* ATCC 33099
*Fusobacterium nucleatum vincentii* ATCC 49256
*Parvimonas micra* ATCC 33270
*Fusobacterium nucleatum polymorphum* ATCC 10953
*Fusobacterium periodonticum* ATCC 33693
*Prevotella intermedia* ATCC 25611 *Streptococcus constellatus* ATCC 27823
Red complex
*Porphyromonas gingivalis* ATCC 33277
*Tannerella forsythia* ATCC 43037
Other
*Streptococcus anginosus* ATCC 33397
*Streptococcus mutans* ATCC 25175
*Selenomonas noxia* ATCC 43541
*Propionibacterium acnes* ATCC 11827
*Gemella morbillorum* ATCC 27824
